# Seroprevalence of Schistosomiasis and Strongyloides infection in HIV-infected patients from endemic areas attending a European infectious diseases clinic

**DOI:** 10.1186/1742-6405-10-23

**Published:** 2013-09-08

**Authors:** Corinna M Sadlier, Aisling Brown, John S Lambert, Gerard Sheehan, Patrick W G Mallon

**Affiliations:** 1 Department of Infectious Diseases, Mater Misericordiae University Hospital, Dublin, Ireland; 2 HIV Molecular Research Group, School of Medicine and Medical Science, University College Dublin, Dublin, Ireland

**Keywords:** Schistosomiasis, Strongyloides, HIV, Eosinophilia

## Abstract

**Background:**

Although the Centres for disease Control and Prevention (CDC) recommends empiric treatment for schistosomiasis and strongyloidiasis (prevalent but treatable parasitic infections) in some refugee groups it is unclear if these guidelines should be extended to non-refugee immigrants from endemic areas. We aimed to assess seroprevalence of, and risk factors for, positive schistosomiasis and strongyloides serology in HIV-infected patients from endemic areas attending a European Infectious Diseases clinic.

**Methods:**

In a prospective cohort study, HIV-infected patients from helminth endemic areas underwent clinical assessment and blood draw for schistosomiasis and strongyloides serology, routine haematology and inflammatory markers (ESR and CRP). Between-group differences were analyzed by Wilcoxin Signed Rank and Fisher’s t tests as appropriate.

**Results:**

Ninety HIV-infected patients (mean [standard deviation (SD)] age 34 [6] years, 29% male) were recruited from May 2008 to June 2009. Nine (10%) subjects tested positive for helminth infections. Seven tested positive for schistosomiasis (8%) while two tested positive for strongyloides (2%). Seropositive subjects were more likely to have higher eosinophil counts (mean [SD]) (0.3 [0.3] vs. 0.15 [0.2] x10^3^cells/cm, *P* = 0.021) with a trend towards lower CD4+ T-cell counts (mean [SD]) (280 [218] vs. 395 [217] cells/mm^3^, *P* = 0.08).

**Conclusion:**

The high prevalence of helminth infections (10%) in asymptomatic HIV infected adults identified in this study supports routine screening of immigrants from helminth endemic areas or with exposure history.

## Introduction

Worldwide over 200 million people are infected with *Schistosoma* species [1] while up to 100 million are infected with *Strongyloides stercoralis*[2]. Areas of highest endemicity include sub-Saharan Africa, eastern South America and regions of Asia. Untreated, both parasites can persist for years to decades and cause significant morbidity and mortality [1,2].

A significant proportion of individuals accessing HIV care in Europe originate from areas endemic for helminth infections. Almost one third of persons accessing HIV care in Ireland are of African origin (*personal communication, Dr Helen Tuite*). These individuals have exposure risk to schistosomiasis, strongyloides and other parasitic infections. Limited data exists on the prevalence of helminth infections in HIV-infected immigrants. European have studies reported a prevalence of schistosomiasis ranging from 11% to 16% and prevalence of strongyloides ranging from 2% to 11% [3-5] in HIV infected immigrants while studies in the United States have reported a prevalence of between 3% and 29% for schistosomiasis and between 0.6% and 26% for strongyloides in HIV infected immigrants[6,7].

Schistosomiasis or bilharzia is caused by a fluke worm of the genus Schistosoma. Adult worms can live for decades in the human host releasing ova causing inflammatory and obstructive disease in the urinary system (*S haematobium*), intestinal disease, hepatosplenic inflammation, and liver fibrosis (*S mansoni*, *S japonicum*) [8]. Immigrants from endemic areas can remain infected for 30 to 40 years [9]. A single dose of praziquantel reliably cures 60 to 90 percent of infected patients and substantially decreases worm burden and egg production in those who are not cured [10].

Strongyloidiasis is caused by infection with *Strongyloides stercoralis* filariform larvae found in soil. Adult worms burrow into the mucosa of the duodenum and jejunum and have a unique capability to replicate inside the host allowing chronic infection to persist for many years and the potential for uncontrolled multiplication. This can result in life-threatening dissemination of larvae in individuals, particularly those with immunodeficiency [11].

Clinical presentations of strongyloidiasis are varied ranging from asymptomatic eosinophilia to hyper-infection with disseminated disease and septic shock in immunocompromised individuals [12,13]. *S. stercoralis* hyperinfection has been described in HIV infected patients as part of the immune reconstitution inflammatory syndrome after starting highly active antiretroviral therapy (HAART) [14,15]. The recommended treatment for strongyloidiasis is ivermectin [16].

The Centers for Disease Control and Prevention (CDC) have published guidelines for screening of refugees for parasitic infections [17] however there are no standards for how or when to look for parasitic infections among non-refugee immigrants from endemic areas that may be resident in a developed country for extended periods. Furthermore there are no guidelines for assessing those from “parasite endemic” regions that are infected with HIV.

The primary objective of this study was to determine the seroprevalence of schistosomiasis and strongyloides among HIV-infected persons that have immigrated to Ireland from “parasite endemic” countries. The secondary objective was to evaluate whether specific symptoms, signs or laboratory parameters are useful in predicting parasitic infection.

## Methods

The Helminth Prevalence Study was a prospective single visit cohort study carried out in the Infectious Diseases clinic at the Mater Misericordiae University Hospital (MMUH) in Dublin from June 2008-June 2009. MMUH is a tertiary referral teaching hospital in a socially deprived area of Dublin and serves a catchment of 185,000 patients.

Consecutive HIV-infected patients from countries of endemic helminth infection were recruited. Patients were screened for the presence of schistosomal and strongyloides antibodies in serum samples using an enzyme-linked immunosorbent assay (ELISA).

The schistosomiasis ELISA used was a schistosomal egg antigen “in house” assay which detects immunoglobulin G antibodies to both *S. mansoni* and *S. haematobium* with high sensitivity and specificity and minimal cross-reactivity. This ELISA is reported to detect 96% of *Schistosoma mansoni* and 92% of *Schistosoma haematobium* infections however, the test does not distinguish active from treated infections [18].

The strongyloides assay used (Bordier Affinity Products SA, Switzerland) detects *strongyloides ratti* somatic larval antigens. Diagnostic accuracy for strongyloides serology has a reported sensitivity of 88%, specificity of 94% and cross reactivity of 77% with other parasitosis, mostly helminthiasis [19,20]. Serological assays were performed at the Hospital for Tropical Diseases, London, the national reference laboratory in the United Kingdom.

Demographic data recorded for all patients included age, gender, country of birth, reason for travel to Ireland, length of stay in Ireland, presence of disease-specific symptoms [21,22], previous diagnosis and treatment of helminth infection and use of HAART. Along with helminth serology, full blood count with differential, C-reactive protein (CRP), erythrocyte sedimentation rate (ESR), lymphocyte subsets and HIV RNA were recorded.

Treatment outcomes and direct stool or urine analysis for parasitic infections were not recorded as this was a single visit study. The patients overseeing health care providers were informed of positive serology and, where necessary, arranged further diagnostics, treatment and follow up.

Data were entered onto study specific case report forms and then transferred to a study specific data base. Continuous variables were analysed using Wilcoxon Signed Rank test and discrete variables by Fisher’s t test as appropriate. All analyses were performed using PASW statistics, version 18 (SPSS, NYC). The study was approved by the local Research Ethics Committee and all patients provided written, informed consent.

## Results

Baseline demographic and disease characteristics are outlined in Table 1. Ninety HIV-infected patients were recruited and tested for the presence of schistosomal and strongyloides antibodies. Mean [SD] age was 34 [6] years. Twenty six (29%) patients were male. Twenty six percent, 31% and 34% were from South, East and West Africa respectively. Sixty eight percent of patients recruited had come to Ireland seeking asylum and 26% for educational purposes.

**Table 1:** Demographics and baseline characteristics of study cohort

	**Cohort**		**Helminth positive**				**P value**
			**Y**		**N**		**(univariate)**
N,%	90		9	10%	81	90%	-
Age, years (mean) [CI]	34	[33–36]	36	[33–39]	34	[33–35]	0.328
Male (N,%)	26	29%	5	56%	21	26%	0.11
Region of Birth (N,%)							
- South Africa	23	26%	4	44%	19	23%	
- East Africa	28	31%	3	33%	25	31%	
- West Africa	31	34%	2	22%	29	36%	
- Central Africa	6	7%	0	0%	6	7%	
- Non-Africa	2	2%	0	0%	2	2%	0.603
Years in Ireland (mean) [SD]	5	[3]	5	[3]	4	[3]	0.59
HIV RNA (median [IQR] (copies/ml)) (n = 89)	50	[1718]	50	[2644]	50	[329779]	0.2
CD4+ T-cell count (mean [CI] (cells/mm3))	398	[345–452]	280	[218]	395	[217]	0.08
Reason for travel to Ireland (N,%)							
- Asylum	61	68%	7	78%	54	67%	
- Education	23	26%	1	11%	22	27%	
- Other	6	7%	1	11%	5	6%	0.53
Previous schistosomiasis therapy (N,%)	6	6.7%	1	1%	5	6%	0.57
Previous strongyloides therapy (N,%)	2	2.2%	1	1%	1	1%	0.15
Eosionophils (x10^3^cells/mm^3^) (mean [CI])	0.16	[0.11-0.2]	0.30	[0.30]	0.15	[0.16]	0.021
Hemoglobin (g/dL) (mean [CI])	12	[12–13]	13	[1]	12	[1]	0.28
MCV (fL) (mean [CI])	84	[82–87]	84	[7]	85	[11]	0.86
CRP (mg/l) (mean [CI]) (n = 79)	5	[3–5]	7(n = 9)	[4]	5(n = 70)	[5]	0.25
ESR (mm/hr) (mean [CI]) (n = 63)	29	[22–37]	22(n = 7)	[28]	31(n = 61)	[28]	0.43
WBC (x10^9^/L) (mean [CI])	5	[4–5]	4	[1]	5	[1]	0.89
Lymphocytes (x10^9^/L) (mean [CI])	2	[1.5-2]	2	[0.6]	2	[0.6]	0.54
Symptoms (N,%)							
- Abdominal pain	7	7.8%	0	0%	7	9%	0.35
- Diarrhoea	1	1%	0	0	1	1%	0.73
- Haematuria	1	1%	0	0	1	1%	0.73
- Dyspnoea	5	6%	0	0	5	6%	0.44
- Wheeze	3	3%	0	0	3	4%	0.55
- Cough	5	6%	1	11%	4	5%	0.44
- Skin eruptions	10	11%	1	11%	9	11%	1
- Any above listed symptom	32	36%	2	22%	30	37%	0.37

*CRP* C-reactive protein, *ESR* erythrocyte sedimentation rate, *MCV* mean corpuscular volume, *N* number, *WCC* white cell count.

Data set was complete for all fields except for ESR and CRP where n = 63 and 79 respectively as outlined in table.

Region of origin was defined according to the *United Nations- Composition of macro geographical (continental) regions, geographical sub-regions, and selected economic and other groupings* (http://unstats.un.org/unsd/methods/m49/m49regin.htm#africa).

Nine (10%) had positive helminth serology (either strongyloides or schistosomiasis). Seven (7.7%) had positive schistosomiasis serology and 2 (2.2%) had positive strongyloides serology. No subject had both positive schistosomiasis and strongyloides serology.

Subjects with positive helminth serology were more likely to have higher eosinophil count (mean [SD]) (0.30 [0.30] versus 0.15 [0.16] ×10^3^cells/mm^3^, P = 0.021) and were noted to have lower CD4+ T-cell counts (mean [SD]) (280 [218] versus 395 [217] cells/mm^3^, P = 0.08).

Although eosinophil count was significantly higher in those with positive helminth serology, only 2 out of 9 (22%) subjects with positive helminth serology had eosinophil counts above the upper limit of normal (0.4 ×10^9^/L).

A single patient with a positive schistosome antibody only, reported previous treatment for both schistosomiasis and strongyloides 20 years earlier in South Africa.

Schistosome antibody positive persons had been resident in Ireland a median of 5 years. Seven (78%) of those with positive helminth serology did not report any relevant symptoms that may have been suggestive of an underlying helminth infection.

## Discussion

This is the first study to report the seroprevalence of schistosomiasis and strongyloides in HIV-infected persons from parasite-endemic regions living in Ireland. Ten percent of our study cohort had serological evidence of infection with schistosomiasis or strongyloides at some time. Sixty eight percent of patients in the study had come to Ireland seeking asylum while 26% had come for educational purposes.

Similar studies carried out in the United Kingdom report a seroprevalence for schistosomiasis alone of 11-17% in HIV-infected African immigrants [3,4]. Mascarello *et al.* report a prevalence of strongyloides infection alone of 11% in 138 HIV-infected adults attending an Italian clinic [5]. Studies carried out in the US looking at helminth infections in general refugee populations have reported a prevalence of 11-70% [23,24].

Identified prevalence in our cohort may be lower than that reported in general refugee populations the US as our patients originated from geographically diverse areas. The US study reporting very high prevalence of helminth infections concentrated on refugees from two specific areas, Somalia and Sudan which may have a greater exposure risk [24].

In addition other studies used a variety of techniques for identifying infection including direct (where the parasite is directly visualized by microscopy) and indirect (where a serological marker of parasitic infection such as antibody is used for diagnosis) methods. Our study looked at serology alone as a marker of helminth infection. Positive serology indicates infection at some time however it does not differentiate between past and active infection.

Direct methods are the most specific means of diagnosing active helminth infection however they are generally regarded as less sensitive as shedding of eggs may be intermittent if patients have a low parasite burden and accuracy is dependent on the observer experience [25,26].

There is some evidence to suggest that HIV-infected individuals may have an impaired serological response which may underestimate infection rates, and although this is not a consistent finding it must be considered when interpreting results in HIV-infected patients [5,27].

Eosinophilia is commonly reported to be associated with schistosomiasis and strongyloides infection including those co-infected with HIV, and may reflect disease activity and the presence of migrating parasites or ova [28]. A number of studies have highlighted the fact that eosinophilia may be absent in helminth infection particularly if infection is chronic [29].

Only 2 of 9 with a positive helminth serology in our study had an eosinophil counts exceeding the upper limit of normal (0.4 × 10^9^/L). A screening approach based on presence of eosinophilia alone would have resulted in missed diagnosis in 7 of the 9 patients with positive helminth serology. In addition, in our study the CD4+ T-cell count of those identified with eosinophilia was not significantly different from those with normal eosinophil count (mean [SD] (324 [262] versus 384 [167], p = 0.37). Presence of eosinophilia has been reported as associated with low CD4+ T-cell count or low nadir CD4+ T-cell count in HIV infection in other studies [30].

Helminth infections are frequently asymptomatic or associated with non specific symptoms and may not be detected unless considered by health care providers and screened for in individuals with exposure history. We did not identify any association between symptoms and positive helminth serology in our study. One of 9 (11%) patients with positive helminth serology reported any potential helminth infection-associated symptom while 37% of those with negative helminth serology reported any symptom.

Increasing evidence suggests that helminth infections may contribute to complications arising from HIV infection. Schistosome co-infection may lower barriers to HIV infection, and contribute to more rapid progression of HIV [31,32]. In addition it has been suggested that HIV vertical transmission may be more likely in helminth-infected mothers [33,34]. Further studies are warranted to clarify these hypotheses, however while waiting for definitive evidence special emphasis should be placed on treatment of schistosomiasis and strongyloides in areas of increased prevalence particularly in HIV endemic populations.

Given the potential morbidity and mortality and the lack of subjective and objective symptoms and signs we suggest that all individuals from endemic areas, particularly those with immunocompromising conditions such as HIV be screened for potential latent parasitic infections.

## Competing interests

CS, AB, GS & JL None. PWGM has received support from the following; Molecular Medicine Ireland, Scientific Foundation Ireland, ViiV Healthcare, Gilead Sciences Ltd., GlaxoSmithKline (Ireland Ltd), Abbott, Merck, Sharp and Dohme and Janssen-Cilag.

## Authors’ contributions

CS carried out data collection, statistical analysis and drafted the manuscript. AB carried out data collection, data entry and helped draft the manuscript. GS helped conceive the study and draft the manuscript. JL helped draft the manuscript. PWGM conceived the study, supervised the statistical analysis and helped draft the manuscript. All authors read and approved the final manuscript.
